# Appendicitis: Its Pathology and Surgery

**Published:** 1901-09

**Authors:** 


**Appendicitis: its Pathology and Surgery.** By CHARLES BARRETT LOCKWOOD. Pp. ix., 287. London: Macmillan and Co., Limited. 1901.

The thoroughness of the author in his well-known methods of aseptic surgery, and his excellent work, in conjunction with Mr. Rolleston, on the anatomy of the vermiform appendix, and the fossæ in its neighbourhood, show him to be well fitted to deal with the subject of appendicitis.

The pathology of this condition, as described in the present volume, leaves little to be desired; but this very fact constitutes at once the strong and the weak points in Mr. Lockwood's method of dealing with the subject.

It has led him to adopt a somewhat lengthy classification of the disease on a pathological basis, which, though scientifically correct, is not very helpful to the clinician, who, when brought in contact with a concrete case, is scarcely capable of distinguishing "appendicitis with ulceration of the mucosa and bacterial invasion," or "appendicitis with lymphangitis and lymphadenitis," from many of the other pathological conditions mentioned by the author; and, moreover, the method of commenting on the clinical history, morbid anatomy, and morbid histology of each of the illustrative cases is, as he himself admits, "tedious and full of repetition."

This is the weak point to which we have referred—the obscuration of the clinical aspect by pathological considerations, and the work is in consequence of more value to the pathologist than to the operating surgeon.

We would, however, bear witness to the painstaking research that is evident throughout the book, which is destined to take a place in the forefront of the voluminous literature of the subject of which it treats.

